# Exploring EPHB4 in gait disorders: VUS detection and phenotypic characterization in a toe-walking cohort

**DOI:** 10.1016/j.gmg.2026.100121

**Published:** 2026-07-14

**Authors:** David Pomarino, Kevin M. Rostásy, Bastian Fregien, Amel Sidi Athmane, Alexander Nazarkin

**Affiliations:** a David Pomarino, Pomarino. Praxis für Ganganomalien, Hamburg, Germany; b Kevin M. Rostásy, Fakultät für Gesundheit (Department für Humanmedizin), Universität Witten/Herdecke, Herdecke, Witten, Germany; c Bastian Fregien, Dr. med, ORTHOMAX, orthopädische üBAG GbR Langenhagen, Germany; d Amel Sidi Athmane, Pomarino. Praxis für Ganganomalien, Hamburg, Germany; e Alexander Nazarkin, Dr. med, Pomarino. Praxis für Ganganomalien, Hamburg, Germany

**Keywords:** Toe walking, EPHB4, Variant of uncertain significance, Pediatric gait disorder, Neuromascular panel, Pes cavus, Next-generation sequencing

## Abstract

**Background:**

Idiopathic toe walking (ITW) is a gait abnormality with a suspected genetic contribution, although its underlying mechanisms remain poorly understood. While ephrin type-B receptor 4 (EPHB4) is primarily recognized for its role in vascular development, this study explored whether EPHB4 variants identified in children with ITW were associated with specific clinical characteristics.

**Methods:**

We retrospectively analyzed the clinical and genetic data of 20 children with toe walking who harbored EPHB4 variants identified using a targeted 49-gene next-generation sequencing (NGS) panel.

**Results:**

Genetic analysis identified 18 distinct EPHB4 variants, all of which were classified as variants of uncertain significance (VUS) according to the five-tier sequence variant classification system. The most common clinical findings were pes cavus (40%), clinodactyly/brachydactyly (40%), and speech or language delay (40%). Notably, only 10% of patients exhibited the venous anomalies typically associated with pathogenic EPHB4 variants.

**Conclusions:**

This study identified a recurrent occurrence of EPHB4 variants in children with idiopathic toe walking. However, these findings should be interpreted with caution because all identified variants were classified as VUS, no clear genotype-phenotype correlation was observed, and ascertainment bias cannot be excluded. Further functional studies and familial segregation analyses are required to determine whether these findings represent a true biological association or incidental observations. These results highlight the complexity of investigating potential genetic contributors to common pediatric gait disorders such as idiopathic toe walking.

**Clinical trial registration:**

not applicable.Retrospective cohort analysis of existing clinical data. Not prospectively registered as no interventions were performed.

## Background

The Eph receptor tyrosine kinase family, the largest subgroup of receptor tyrosine kinases (RTKs), plays a central role in cell-cell communication, tissue patterning, and embryonic development through interactions with membrane-bound ephrin ligands. [1], [2], [3], [4] Based on ligand specificity and structural characteristics, Eph receptors are classified into two subclasses: EphA receptors, which bind ephrin-A ligands, and EphB receptors, which bind ephrin-B ligands. [5] Among these receptors, ephrin type-B receptor 4 (EPHB4) is distinguished by its essential role in vascular development and its emerging significance in oncology. [6], [7], [8], [9].

EPHB4, a member of the EphB subclass, preferentially binds ephrin-B2 and mediates bidirectional signaling involved in the regulation of cell adhesion, migration, and tissue boundary formation. [3], [10] Structurally, EPHB4 consists of an extracellular ephrin-binding domain, a transmembrane region, and an intracellular tyrosine kinase domain that activates downstream signaling pathways, including PI3K/AKT and MAPK. [11], [12] Notably, EPHB4 is essential for venous specification during embryogenesis, where its interaction with epherin-B2 establishes arterial-venous differentiation [13]. Beyond its developmental functions, EPHB4 has increasingly been implicated in human disease, particularly in cancer progression and pathological angiogenesis. Germline EPHB4 variants have also been associated with several vascular disorders, including capillary malformation-arteriovenous malformation syndrome (CM-AVM), [14], [15] vein of Galen aneurysmal malformation, [16] and venous valve aplasia. [17] In oncology, the interaction between EPHB4 and ephrin-B2 has been reported to exert both tumor-promoting and tumor-suppressive effects, reflecting the complex and context-dependent nature of EPHB4 signaling. [18], [19], [20], [21] Consequently, EPHB4 has attracted considerable interest as a potential therapeutic target.

In addition to its vascular and oncologic roles, emerging evidence suggests that EPHB4 may contribute to neural development. In vitro studies have demonstrated that EPHB4 regulates the self-renewal, proliferation, and neuronal differentiation of human embryonic neural stem cells through the Abl-Cyclin D1 signaling pathway. [22].

Idiopathic toe walking is a gait pattern with a suspected genetic contribution. [23] As a clinical sign, it has been reported in several hereditary and neurological disorders, including Charcot-Marie-Tooth disease, cerebral palsy, and autism spectrum disorder. [24], [25], [26] It is defined as persistent walking on the forefoot beyond two to three years of age in the absence of an identifiable neurological or orthopedic cause.^25^, [27] In a previous study of children with persistent non-idiopathic toe walking (n-ITW), we investigated potential genetic causes using a targeted 49-gene next-generation sequencing (NGS) panel. The genes included in this panel have all been associated with hereditary neuromuscular disorders that may present with gait abnormalities.^23^.

During this targeted sequencing analysis, heterozygous EPHB4 variants were identified in a subset of patients, despite EPHB4 not previously being associated with gait disorders. In the present study, we describe the clinical characteristics of these individuals as an exploratory analysis intended to generate hypotheses for future studies investigating a potential association between EPHB4 and toe walking.

## Materials and Methods

This retrospective four-year study investigated potential genetic contributors to persistent idiopathic toe walking (ITW) in pediatric patients referred for gait analysis. Eligible participants met the following inclusion criteria: 1- Parent-reported toe walking for more than 50% of the day. 2- Completion of a dedicated 49-gene next-generation sequencing (NGS) panel. 3- Absence of diagnosed neurological disorders (e.g., cerebral palsy, tethered cord syndrome, or autism spectrum disorder) and significant musculoskeletal abnormalities, including limb length discrepancy, lower-limb axis deformities, scoliosis, or severe foot deformities.

All participants underwent a standardized clinical assessment consisting of a detailed medical history questionnaire and physical examination. The clinical and anamnestic parameters systematically recorded at our institution are summarized in Table 1.

**Table 1:** Clinical and Anamnestic Parameters Assessed in Patients.

**Patient History**	• Perinatal history • Age of onset of TW • Onset of walking (OoW) • Family history of toe walking or related neuropathies • Speech and language difficulties (e.g., dysarthria)^1^ • Social development^1^ • Visual impairments^1^ • Pain localization^1^ • Muscle-related symptoms (myalgia, cramps, fatigue)^1^ • Comorbidities
**Toe walking Diagnosis**	• Visual gait analysis • Ankle joint mobility^2^ • Ability to balance on heels • Heel walking test • Foot drop test (toe lifting)
**Physical Symptoms and Abnormalities**	• Spinal abnormalities (lumbar spine hyperlordosis)^3^ • Structural abnormalities (pes cavus, clinodactyly, brachydactyly, pectus excavatum)^3^ • Reflex anomalies^4^ • Presence of tremor^5^

*Notes: ¹Parent/Caregiver-reported data; ²Ankle mobility assessed via goniometry (neutral/0° method; restricted = dorsiflexion <20° or plantarflexion <50°); ³Morphological inspection; ⁴Patellar reflex evaluation; ⁵Tremor assessed with arms extended forward (palms down), provoked by repeated hand opening-closing; rhythmic oscillations post-movement were considered positive.*.

### Statistical Analysis

Given the exploratory nature of this retrospective cohort study, statistical analyses were limited to descriptive statistics. All analyses were performed using LibreOffice Calc.

Descriptive statistics were used to characterize the study population, including age distribution, sex distribution, and variant classification frequencies, as well as to determine the prevalence of clinical features such as musculoskeletal abnormalities, developmental characteristics, vascular findings, and family history.

Categorical variables are presented as absolute numbers (n) and percentages of the total cohort (n/N × 100). Continuous variables are presented as mean ± standard deviation (SD) and range (minimum–maximum).

No comparative statistical analyses were performed because the study lacked a control group, all identified variants were classified as variants of uncertain significance (VUS), and the study was designed as a hypothesis-generating exploratory investigation.

### Genetic Analysis

Patients underwent genetic testing using a targeted 49-gene next-generation sequencing (NGS) panel developed to identify hereditary neuropathies and myopathies potentially associated with toe walking. Individuals investigated using alternative genetic approaches, such as whole-exome sequencing, were not included. Genetic testing was performed as part of routine clinical diagnostics using saliva samples collected during standard patient care. The targeted NGS panel, developed based on genes associated with relevant Human Phenotype Ontology (HPO) terms, analyzed 49 neuromuscular-related genes. Sequencing was performed on the Ion Torrent platform and included all coding exons together with adjacent intronic regions (±10 bp). Sequence variants were classified according to the five-tier variant classification system described by Plon et al. [28], comprising pathogenic (class 5), likely pathogenic (class 4), variant of uncertain significance (class 3), likely benign (class 2), and benign (class 1) variants. Only pathogenic, likely pathogenic, and variants of uncertain significance were included in the present analysis, whereas benign and likely benign variants were excluded but remained available upon request. Variant interpretation was supported by in silico prediction tools, including MutationTaster, PolyPhen−2, and Mutation Assessor, together with publicly available databases such as HGMD Professional, ClinVar, LOVD, and dbSNP. The analytical sensitivity of the NGS panel for clinically relevant variants exceeded 96%.

## Results

### Study Population

Among 1300 pediatric patients evaluated for toe walking, 20 individuals were identified as heterozygous carriers of EPHB4 variants. The final study cohort comprised 10 males and 10 females with a mean age of 7 years (range, 2–17 years).

### Genetic Testing results

All 20 patients harbored variants of uncertain significance (VUS) in EPHB4, representing 18 distinct sequence variants. Variant classification was performed according to the five-tier classification system described by Plon et al., based on the clinical evidence and interpretation standards available at the time each laboratory report was issued. Variant interpretation incorporated evidence from biomedical literature, population databases (ClinVar, gnomAD, and HGMD), and in silico prediction tools (PolyPhen−2, SIFT, and MutationTaster). Table 2 summarizes the identified variants together with their reported population frequencies and report dates.

**Table 2:** Summary of EPHB4 Variants and Population Frequencies in Study Cohort.

**Patient ID**	**Age**	**Gender**	**Variant c. HGVS**	**Frequencies**	**Report date**
1	2	m	c.1085 G>A	0. 0029%	17.03.2021
2	2	m	c.1144 G>A	0.0602%	10.10.2022
3	13	m	c.118 G>A	0.0012%	10.08.2022
4	9	m	c.1205 A>G	Not available	25.08.2023
5	17	f	c.1295 A>T	Not available	13.07.2022
6	3	f	c.1429 G>A	0.0056%	10.02.2023
7	6	f			10.09.2021
8	7	f	c.1561 G>A	Not available	27.11.2020
9	5	m	c.1692–6 C>A	0.0652%	09.08.2022
10	3	m	c.1905G>T	0.0044%	01.09.2023
11	7	m	c.236 C>T	0.012%	27.11.2020
12	5	f	c.2417 A>T	Not available	21.06.2023
13	11	m	c.2596 C>T	0.0004%	16.07.2024
14	10	f	c.280 A>G	0.0016%	06.06.2024
15	5	m	c.2826 C>G	Not available	04.11.2021
16	8	f	c.691 C>T	0,02%	14.06.2021
17	3	f			19.01.2021
18	7	f	c.703 C>T	0.154%	18.11.2020
19	7	f	c.817 C>A	Not available	21.12.2022
20	4	f	c.1405 G>A	0.01%	25.02.2019

**Clinical Findings** Patients exhibited a broad spectrum of clinical findings. Muscle pain involving the back, knees, calves, legs, feet, or heels was reported by six patients. Four patients experienced muscle fatigue, whereas one reported muscle cramps. Physical examination revealed pes cavus in eight patients and clinodactyly and/or brachydactyly in eight patients. Pectus excavatum was observed in three individuals, while only one patient demonstrated an essential tremor during provocation testing. Lumbar hyperlordosis was present in one patient. A positive family history of similar symptoms was documented in four cases (two paternal, one maternal, and one sibling). Speech or language difficulties were reported by parents in eight patients.

## Discussion

The identification of EPHB4 variants of uncertain significance (VUS) in this cohort of children with toe walking represents an observation that warrants further investigation. Although EPHB4 is primarily recognized for its role in vascular development, its identification in this cohort raises the possibility of previously unrecognized biological functions. Nevertheless, the observed clinical findings should be interpreted cautiously, and no causal relationship can be inferred from the present data.

## Clinical Context

The clinical features observed in our cohort, including pes cavus, tremor, and speech or language delay, are not specific to EPHB4-related disorders and may reflect other genetic or developmental conditions. In contrast, pathogenic EPHB4 variants are typically associated with vascular abnormalities. One possible explanation is that venous malformations involving the calf musculature may alter gait biomechanics. Several case reports have described toe walking secondary to intramuscular venous malformations, including one adolescent with unilateral followed by bilateral toe walking associated with an intramuscular venous malformation identified by magnetic resonance imaging and three additional patients presenting with equinus deformity associated with vascular anomalies. [29], [30].

These reports suggest that structural venous abnormalities may contribute to altered gait through muscle contracture. Consistent with this hypothesis, one patient carrying the c.1405 G>A variant presented with unilateral toe walking secondary to right-sided Achilles tendon shortening and gastrocnemius-soleus contracture in the presence of a confirmed ipsilateral venous malformation infiltrating the calf musculature (Fig. 1). Clinical findings included persistent unilateral equinus gait (right greater than left), inability to achieve heel strike or neutral ankle dorsiflexion with the knee extended, asymmetric calf involvement with palpable firmness and functional impairment, and bilateral vascular malformations (predominantly right-sided) previously treated with image-guided sclerotherapy.

**Fig. 1:**
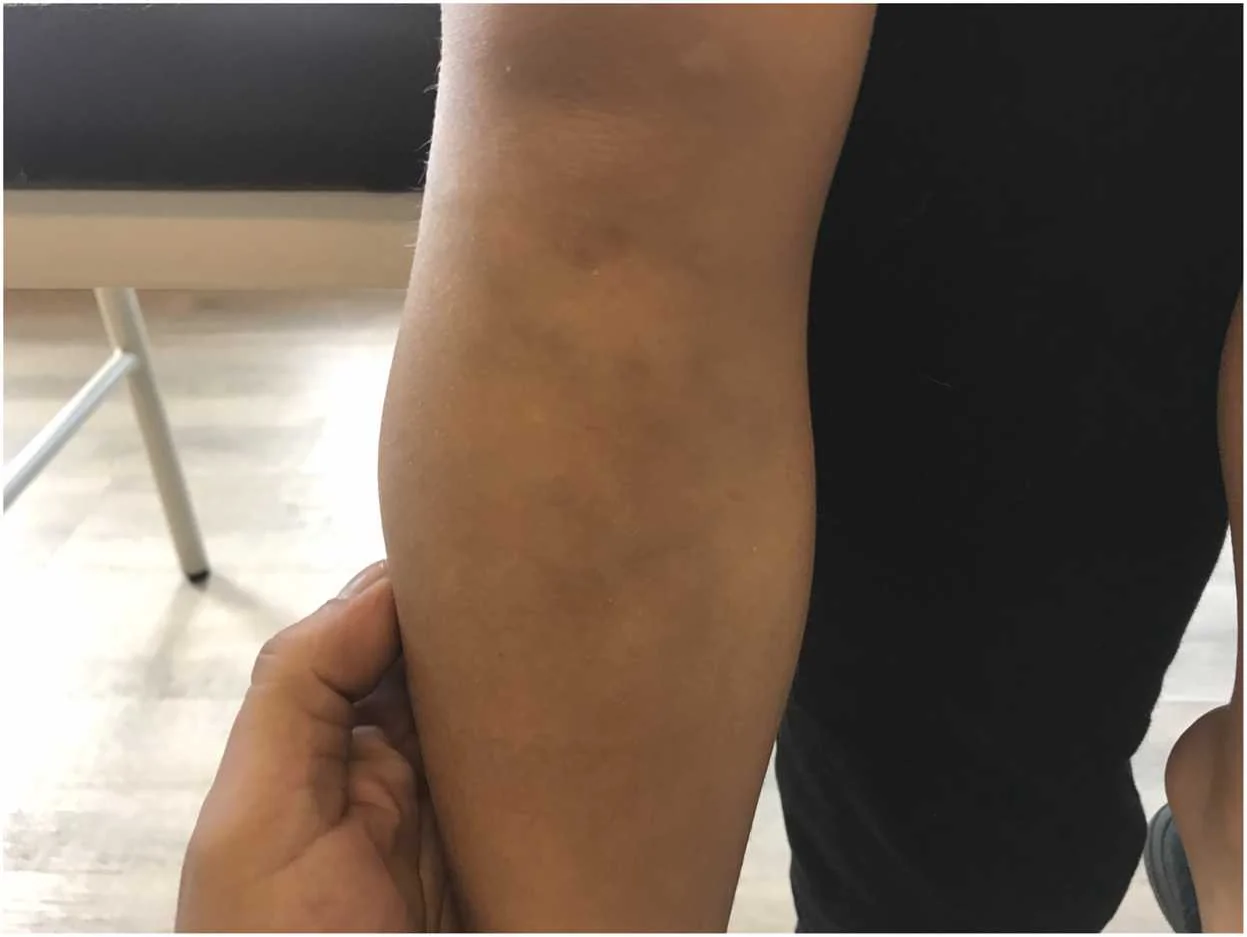
Right calf venous malformation with associated equinus gait.

A second patient carrying the c.1085 G>A variant also demonstrated unilateral calf asymmetry and prominent plantar venous markings suggestive of underlying vascular involvement (Fig. 2, Fig. 3).

**Fig. 2:**
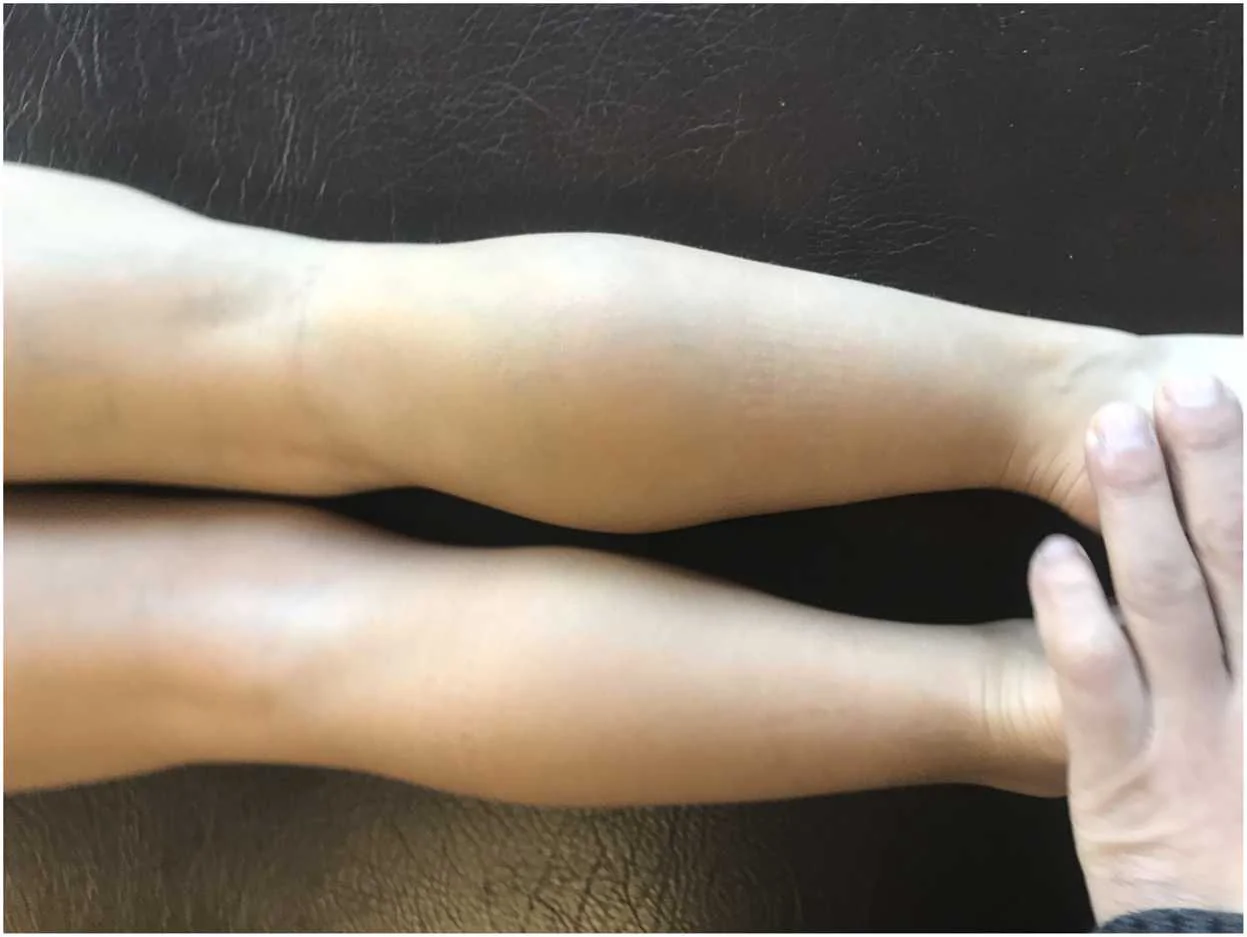
Asymmetric calf contour in a child with toe walking.

**Fig. 3:**
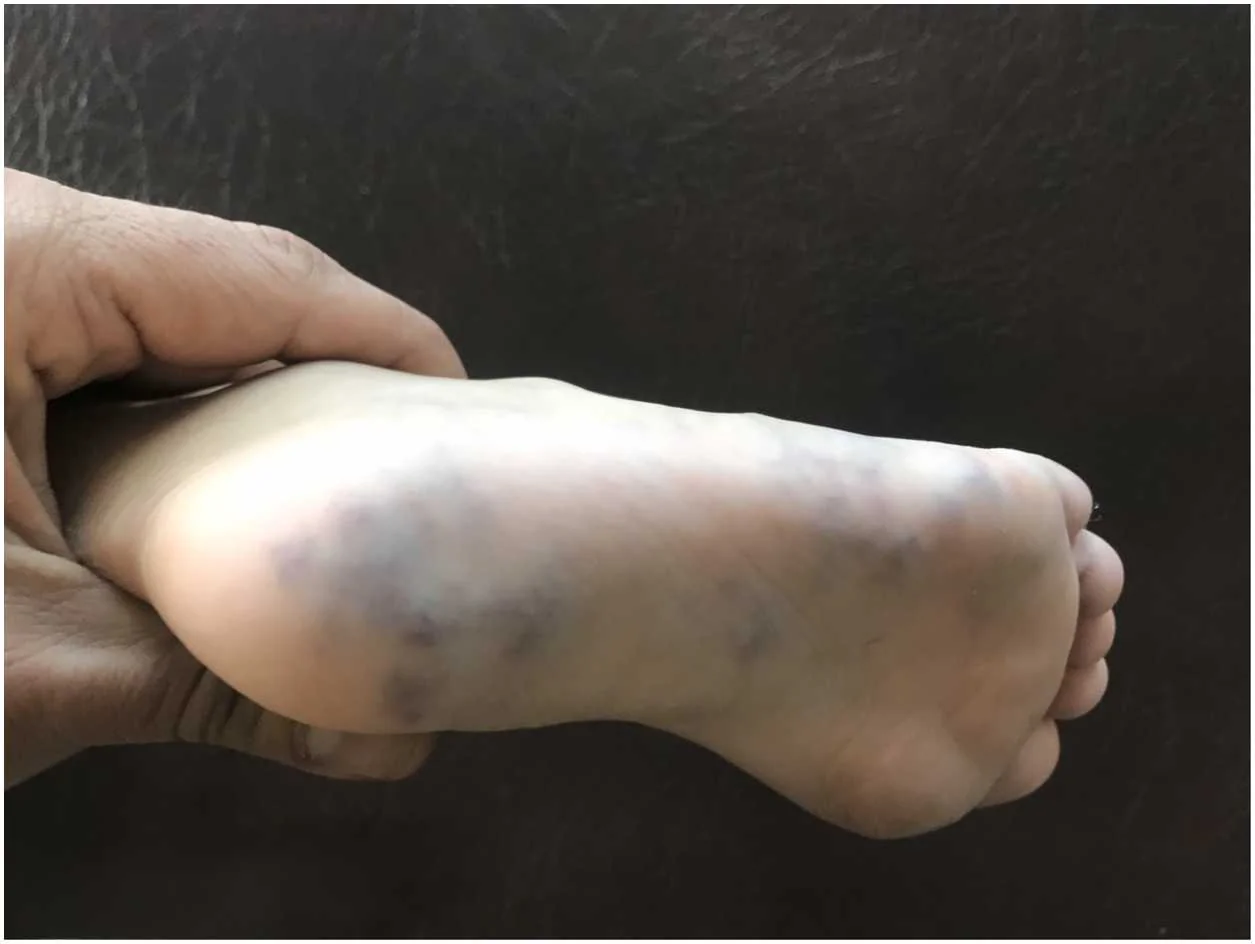
Plantar venous markings in a child with toe walking.

Text 1: Clinical photograph showing anteroposterior cutaneous discoloration.

Text 2: *Clinical image showing left-right asymmetry in calf musculature. Such changes may suggest underlying venous malformation potentially affecting gait biomechanics.*

Text 3: Prominent subcutaneous venous patterning on the plantar surface, possibly reflecting venous ectasia or malformation.

### Genetic Interpretation

Because all identified EPHB4 variants were classified as variants of uncertain significance, their clinical relevance remains uncertain. According to current standards for sequence variant interpretation, VUS require additional evidence, including familial segregation studies, functional validation, or consistent recurrence in affected individuals before pathogenicity can be established. Although the repeated identification of EPHB4 VUS in our cohort is noteworthy, these findings alone are insufficient to support a causal relationship. [31].

### Biological Plausibility

Although EPHB4 is primarily involved in vascular development, its potential contribution to neuromuscular function may be indirect through effects on vascular perfusion, vascular smooth muscle physiology, or local blood flow regulation. The established role of EPHB4 in vascular smooth muscle contractility [32] provides biological plausibility for this hypothesis; however, no direct involvement in motor neuron development or skeletal muscle development has yet been demonstrated. Future studies should therefore include expression analyses in neural and muscular tissues, functional characterization of individual variants, and familial segregation studies to clarify whether EPHB4 contributes to the pathophysiology of toe walking.

## Limitations

This study has several limitations. First, all identified EPHB4 variants were classified as variants of uncertain significance, precluding conclusions regarding pathogenicity in the absence of functional validation or segregation analyses. Second, the relatively small cohort limited the ability to identify robust genotype-phenotype relationships. Third, the retrospective study design relied on existing clinical records, which may have resulted in variable phenotypic characterization, underrecognition of subtle vascular abnormalities, and incomplete family data. Technical limitations of the targeted 49-gene NGS panel should also be considered. The panel did not evaluate non-coding regions, copy number variants, repeat expansions, or low-level mosaicism and was restricted to genes already implicated in neuromuscular disorders, potentially overlooking novel disease-associated genes. Furthermore, the overlap of clinical findings with hereditary neuropathies, such as pes cavus and speech delay, complicates differentiation between potential EPHB4-related manifestations, coexisting undiagnosed disorders, and idiopathic presentations. Finally, the absence of an unaffected control group precludes assessment of whether the frequency of EPHB4 VUS differs from that expected in the general population.

## Conclusion

This study reports the incidental identification of EPHB4 variants of uncertain significance in a selected cohort of children with toe walking. Although the recurrence of these variants across multiple patients is noteworthy, their classification as variants of uncertain significance and the absence of characteristic vascular phenotypes in most patients—except for two cases—preclude any conclusions regarding causality. The clinical features observed among variant carriers overlap with those seen in hereditary neuropathies and connective tissue disorders, requiring cautious interpretation. Emerging evidence supporting a role for EPHB4 in neural development provides a biologically plausible, although currently unproven, basis for further investigation. Functional studies and familial segregation analyses will be essential to determine whether EPHB4 contributes to the pathophysiology of toe walking. Until such evidence is available, the present findings should be regarded as exploratory and hypothesis-generating rather than demonstrative of a clinical association. Validation in independent cohorts, together with functional characterization of the identified variants, will be necessary to determine whether these observations reflect a true biological association or are the result of ascertainment bias.

## List of abbreviations

ITW = Idiopathic toe walking / NGS = Next-Generation Sequencing / EPHB4 = Ephrin type-B receptor 4 / VUS = Variants of uncertain significance/ P = Pathogenic / LP = Likely pathogenic /ACMG = American College of Medical Genetics and Genomics / OoW = Onset of walking / HPO = Human Phenotype Ontology / VM = Venous malformation.

## Ethics approval and consent to participate

The study was conducted in accordance with the Declaration of Helsinki and approved by the ethical board of Deutschen Verbandes für Physiotherapie an der Physio-Akademie in Wremen, Germany, project number 2025–02. For participants under 16 years of age, written informed consent was obtained from their parents or legal guardians, and all data were anonymized.

## Authors’ contributions

D.P. conceived the study, collected clinical data, performed genetic analysis, and drafted the manuscript.

K.R. critically revised the manuscript for intellectual content and methodology.

B.F. assisted with clinical interpretation and literature review.

J.O.S. contributed to data organization and preliminary analysis.

A.Z. provided technical support for work design.

All authors reviewed and approved the final manuscript.

## Consent for publication

Not applicable (no individual patient images or identifiable data are included).

## Funding

This research received no specific grant from funding agencies in the public, commercial, or not-for-profit sectors.

## Data Availability

The datasets generated during this study (variant classifications and clinical phenotypes) are included in the manuscript. Raw sequencing data may be made available from the corresponding author upon reasonable request. The authors declare that they have no competing interests.

## References

[1] Pasquale E.B. (2008). Eph-ephrin bidirectional signaling in physiology and disease. Cell.

[2] Flanagan J.G., Vanderhaeghen P. (1998). The ephrins and Eph receptors in neural development. Annu. Rev. Neurosci..

[3] Pasquale E.B. (2005). Eph receptor signalling casts a wide net on cell behaviour [published correction appears in Nat Rev Mol Cell Biol. 2005 Jul;6(7):589]. Nat. Rev. Mol. Cell. Biol..

[4] Parrinello S., Napoli I., Ribeiro S. (2010). EphB signaling directs peripheral nerve regeneration through Sox2-dependent Schwann cell sorting. Cell.

[5] (1997). Unified nomenclature for Eph family receptors and their ligands, the ephrins. Eph Nomencl. Comm. Cell..

[6] Lv J., Xia Q., Wang J., Shen Q., Zhang J., Zhou X. (2016). EphB4 promotes the proliferation, invasion, and angiogenesis of human colorectal cancer. Exp. Mol. Pathol..

[7] Lafleur K., Huang D., Zhou T., Caflisch A., Nevado C. (2009). Structure-based optimization of potent and selective inhibitors of the tyrosine kinase erythropoietin producing human hepatocellular carcinoma receptor B4 (EphB4). J. Med. Chem..

[8] Wu Q., Suo Z., Kristensen G.B., Baekelandt M., Nesland J.M. (2006). The prognostic impact of EphB2/B4 expression on patients with advanced ovarian carcinoma. Gynecol. Oncol..

[9] Alam S.M., Fujimoto J., Jahan I., Sato E., Tamaya T. (2009). Coexpression of EphB4 and ephrinB2 in tumor advancement of uterine cervical cancers. Gynecol. Oncol..

[10] Davy A., Soriano P. (2005). Ephrin signaling in vivo: look both ways. Dev. Dyn..

[11] Li G.X., Ma B., Zhang S. (2025). EphB4-ephrin-B2 are targets in castration-resistant prostate cancer. Br. J. Cancer.

[12] Chen D., Van der Ent M.A., Lartey N.L., King P.D. (2023). EPHB4-RASA1-Mediated Negative Regulation of Ras-MAPK Signaling in the Vasculature: Implications for the Treatment of EPHB4- and RASA1-Related Vascular Anomalies in Humans. Pharm. (Basel).

[13] Wang H.U., Chen Z.F., Anderson D.J. (1998). Molecular distinction and angiogenic interaction between embryonic arteries and veins revealed by ephrin-B2 and its receptor Eph-B4. Cell.

[14] Amyere M., Revencu N., Helaers R. (2017). Germline Loss-of-Function Mutations in EPHB4 Cause a Second Form of Capillary Malformation-Arteriovenous Malformation (CM-AVM2) Deregulating RAS-MAPK Signaling. Circulation.

[15] Bayrak-Toydemir P., Stevenson D.A., Adam M.P., Feldman J., Mirzaa G.M., Pagon R.A., Wallace S.E., Amemiya A. (February 22, 2011). GeneReviews®. Seattle (WA).

[16] Vivanti A., Ozanne A., Grondin C. (2018). Loss of function mutations in EPHB4 are responsible for vein of Galen aneurysmal malformation. Brain.

[17] Lyons O., Walker J., Seet C. (2021). Mutations in EPHB4 cause human venous valve aplasia. JCI Insight.

[18] Noren N.K., Pasquale E.B. (2007). Paradoxes of the EphB4 Receptor in Cancer. Cancer Res..

[19] Sagar V., Vatapalli R., Lysy B. (2019). EPHB4 inhibition activates ER stress to promote immunogenic cell death of prostate cancer cells. Cell. Death Dis..

[20] Chen Y., Zhang H., Zhang Y. (2019). Targeting receptor tyrosine kinase EphB4 in cancer therapy. Semin. Cancer Biol..

[21] Salgia R., Kulkarni P., Gill P.S. (2018). EphB4: A promising target for upper aerodigestive malignancies. Biochim. Biophys. Acta Rev. Cancer.

[22] Liu T., Zeng X., Sun F. (2017). EphB4 Regulates Self-Renewal, Proliferation and Neuronal Differentiation of Human Embryonic Neural Stem Cells in Vitro. Cell. Physiol. Biochem..

[23] Pomarino D., Emelina A., Heidrich J. (2023). NGS-Panel Diagnosis Developed for the Differential Diagnosis of Idiopathic Toe Walking and Its Application for the Investigation of Possible Genetic Causes for the Gait Anomaly. Glob. Med. Genet..

[24] Mary P., Servais L., Vialle R. (2018). Neuromuscular diseases: Diagnosis and management. Orthop. Traumatol. Surg. Res..

[25] Pernet J., Billiaux A., Auvin S. (2010). Early onset toe-walking in toddlers: a cause for concern?. J. Pediatr..

[26] Camia Michela, Sacco Roberto, Boncoddo Maria, Bellomo Fabiana, Cucinotta Francesca, Ricciardello Arianna, Turriziani Laura, Tomaiuolo Pasquale, Costantini Riccardo Cuoghi, Persico Roberto D.'Amico, Antonio M. (2024). Toe walking in children and adolescents with Autism Spectrum Disorder: Relationship with sensory and motor functions,language, cognition, and autism severity. Res. Autism Spectr. Disord..

[27] Engelbert R., Gorter J.W., Uiterwaal C., van de Putte E., Helders P. (2011). Idiopathic toe-walking in children, adolescents and young adults: a matter of local or generalised stiffness?. BMC Musculoskelet. Disord..

[28] Plon S.E., Eccles D.M., Easton D., Foulkes W.D., Genuardi M., Greenblatt M.S., Hogervorst F.B., Hoogerbrugge N., Spurdle A.B., Tavtigian S.V. (2008 Nov). IARC Unclassified Genetic Variants Working Group. Sequence variant classification and reporting: recommendations for improving the interpretation of cancer susceptibility genetic test results. Hum. Mutat..

[29] Babiker Mohamed O.E., Yeo T.H., Goodwin S. (2015). A Venous Malformation Presenting as Toe Walking. Pediatr. Neurol..

[30] Domb B.G., Khanna A.J., Mitchell S.E., Frassica F.J. (2004). Toe-walking attributable to venous malformation of the calf muscle. Clin. Orthop. Relat. Res..

[31] Richards S., Aziz N., Bale S. (2015). Standards and guidelines for the interpretation of sequence variants: a joint consensus recommendation of the American College of Medical Genetics and Genomics and the Association for Molecular Pathology. Genet. Med..

[32] Wang Y., Thorin E., Luo H. (2015). EPHB4 Protein Expression in Vascular Smooth Muscle Cells Regulates Their Contractility, and EPHB4 Deletion Leads to Hypotension in Mice. J. Biol. Chem..

